# Tailored Revascularization and Endovascular Strategy in the Management of Type 1A Endoleak After Descending Thoracic Aortic Aneurysm (TAA) Repair: A Case Report

**DOI:** 10.7759/cureus.88153

**Published:** 2025-07-17

**Authors:** Roberto A Martin, Julian Scaccia, Pranav S Tadepalli, Stephanie Yakoubovitch, Michael A Lopez, Miguel Lopez-Viego

**Affiliations:** 1 Dr. Kiran C. Patel College of Osteopathic Medicine, Nova Southeastern University, Fort Lauderdale, USA; 2 Surgery, Charles E. Schmidt College of Medicine, Florida Atlantic University, Boca Raton, USA; 3 Cardiothoracic Surgery, UTHealth Houston, Houston, USA; 4 Surgery, Bethesda Hospital, Boynton Beach, USA

**Keywords:** balloon-assisted fixation, carotid-carotid bypass, carotid-subclavian transposition, complex aortic arch anatomy, endovascular reintervention, hybrid surgical approach, retroesophageal tunnel, thoracic aortic aneurysm (taa), thoracic endovascular aortic repair (tevar), type 1a endoleak

## Abstract

Thoracic endovascular aortic repair (TEVAR) has emerged as the preferred treatment for descending thoracic aortic aneurysms (TAA), but one of its major complications, type 1A endoleaks, can lead to aneurysm rupture and mortality if not managed promptly and effectively. This case report details an 87-year-old male patient who developed a type 1A endoleak following a TEVAR procedure for a large descending TAA. The patient's complex vascular anatomy, including a dominant right vertebral artery and an aneurysmal left subclavian artery, necessitated a tailored surgical approach. A right-to-left carotid bypass via a retroesophageal tunnel and left carotid to left subclavian transposition were performed, followed by TEVAR with balloon-assisted fixation to resolve the endoleak. This case underscores the importance of a strategic, individualized surgical approach in managing post-TEVAR complications, particularly in high-risk patients. This report highlights the effectiveness of hybrid surgical approaches and less invasive interventions, such as balloon-assisted fixation, to optimize patient outcomes while reducing procedural complexity and risk.

## Introduction

Thoracic aortic aneurysm (TAA) is considered a medical emergency, defined as a 50% increase in the wall diameter of the artery. TAAs arise from degeneration of the aortic media, typically due to structural breakdown of elastin and collagen within the aortic wall. This process is influenced by inflammatory pathways involving transforming growth factor-B (TGF-B), which activate matrix metalloproteinases and cathepsins, driving the destruction of this elastic layer. The compromised elasticity leads to increased arterial wall compliance, ultimately resulting in progressive aortic dilation, especially in the setting of chronic hypertension or other hemodynamic stressors [1]. TAA is becoming more prevalent due to the increased detection rate by advanced imaging techniques available today [2]. The risk of TAA rupture is significant, occurring at a yearly rate of 3.7% [3], with an even greater risk in elderly patients with multiple comorbidities. Surgical treatment of TAA can be done via open repair or an endovascular route, with the latter demonstrating a significant reduction in mortality, morbidity, and length of stay. Characterized by the insertion of an endovascular stent graft and creation of a carotid-subclavian bypass, thoracic endovascular aortic repair (TEVAR) has become the preferred intervention for treating descending TAA, offering a minimally invasive alternative which is advantageous for a few key reasons: absence of a thoracotomy scar, decreased need for mechanical circulatory support, and a lack of need to cross clamp the aorta [4].

One of the most significant complications of TEVAR is an endoleak, defined as persistent blood flow outside the stent graft and into the aneurysm sac. Endoleaks are classified into five types based on the source of the vessels that cause the inflow into the aneurysm sac: type 1A, inadequate seal at the proximal graft attachment site; type 1B, leak at the distal attachment site; type 2, retrograde flow from collateral vessels; type 3, modular disconnection or fabric disruption in the graft; type 4; graft porosity; and type 5, enlarging aneurysm sac without visible endoleak (also known as endotension) [5].

Type 1A endoleaks are particularly high risk due to their direct communication with systemic arterial pressure, which maintains pressurization of the aneurysm sac and increases the likelihood of rupture. They present an overall mortality rate of 50% and an operative mortality rate of 41% [6]. In patients with complex aortic arch anatomy or insufficient proximal landing zones, traditional endovascular solutions may be inadequate. In such cases, hybrid approaches combining open surgical revascularization with endovascular repair are increasingly utilized to optimize outcomes.

This case report describes a patient with a type 1 endoleak following TEVAR, requiring carotid-subclavian bypass and proximal stent graft extension, highlighting the role of individualized strategies in managing complex post-TEVAR complications. This report serves to highlight strategic surgical planning to ensure adequate endograft fixation and to explore the effectiveness of alternative revascularization techniques in optimizing patient outcomes.

## Case presentation

**Figure 1:**
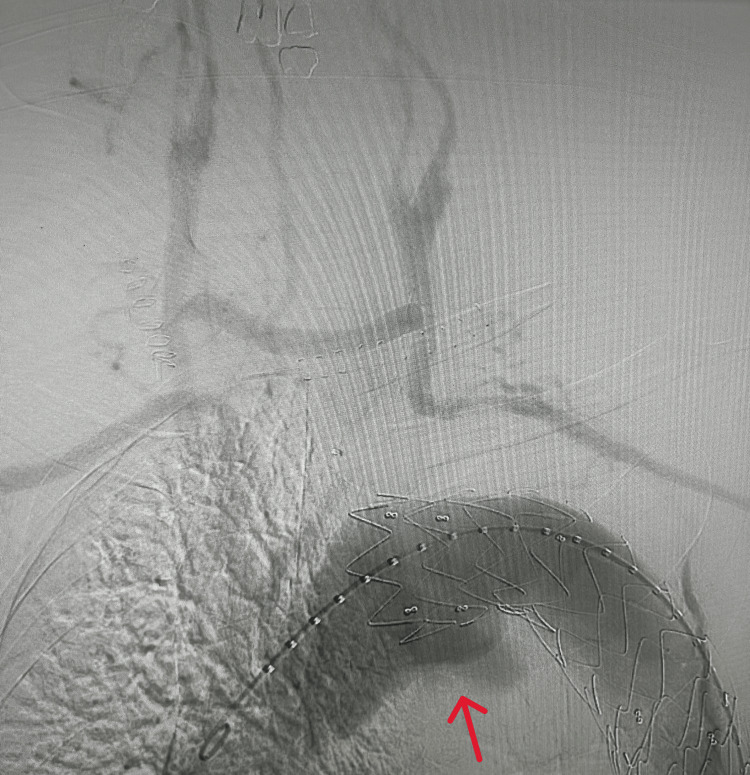
Endoleak at proximal attachment site after TEVAR TEVAR: thoracic endovascular aortic repair

An 87-year-old male patient with a complex medical history initially presented to our hospital with lower back pain. His medical history was notable for a TAA status post-endovascular stent graft (three years prior), hypertension (HTN), hyperlipidemia (HLD), abdominal aortic aneurysm (AAA), and chronic obstructive pulmonary disease (COPD) with a long smoking history. Computed tomography angiography (CTA) of the head and neck revealed a descending TAA just below the arch measuring up to 9.2 cm with a large aneurysmal sac (dramatic interval increase from the previous 7.1 cm measurement), aneurysmal dilation of the left subclavian artery with a diameter of 4.0 cm. CTA of the chest and abdomen revealed the previously placed aortic stent beginning distal to the left subclavian artery and a dominant right vertebral artery. There was a large endoleak extending inferiorly into the aneurysmal sac measuring over 4 cm in diameter, appearing to be a type 1A endoleak associated with the proximal attachment site (Figure 1). No retroperitoneal hemorrhage due to the leak was seen. The patient was experiencing an exacerbation of emphysema at this time. The diagnosis of status post-TEVAR for TAA with type 1A endoleak with 9.3 cm diameter was made, and the following procedures were conducted: right-to-left carotid bypass, left carotid to left subclavian transposition, and completion angiogram through the right common carotid.

A right-to-left retroesophageal carotid-carotid bypass was performed using a ringed 8 mm Propaten graft. The graft was then anastomosed end-to-side to the left common carotid artery. The left common carotid artery was transected at its origin from the aortic arch, and an end-to-end anastomosis was created between the distal left common carotid and the left subclavian artery proximal to the left vertebral artery. Hemostasis was secured, a drain was placed, and the wounds were closed. The patient was extubated and transferred to the PACU in stable condition. 

**Figure 2:**
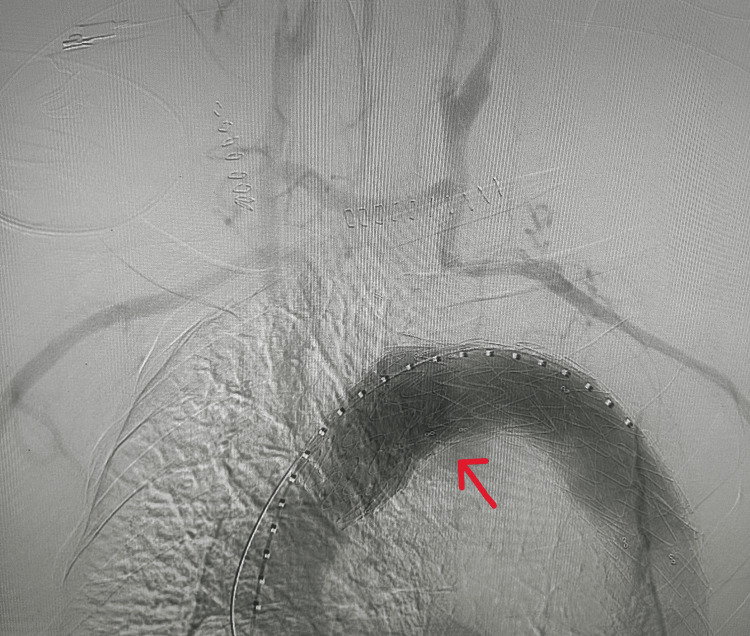
Final image after extra-anatomical reconstruction with proximal cuff extension

The following day, the patient underwent a TEVAR procedure. Bilateral femoral artery access was obtained, and a 24-French sheath was advanced into the descending thoracic aorta. A Gore TAG thoracic stent graft was deployed in the aortic arch, just beyond the origin of the innominate artery (zone 2), ensuring full patency of the innominate artery. Post-deployment angiography initially showed a type 3 endoleak at the graft junction, which was successfully resolved with post-deployment balloon dilation. Final angiography confirmed excellent graft positioning, no endoleak, and patent flow through the graft, innominate artery, right common carotid, right-to-left carotid bypass, and left subclavian artery (Figure 2). Hemostasis was achieved at the femoral access sites using the Perclose ProGlide closure device, and the patient was transferred to the ICU in stable condition.

## Discussion

Post-TEVAR complications such as type 1A endoleaks require an individualized approach tailored to the patient's vascular anatomy and overall health. In this case, the patient presented with a significant 9.3 cm TAA and a type 1A endoleak following a prior TEVAR. The presence of a dominant right vertebral artery necessitated preservation of the right-sided cerebral perfusion, limiting the feasibility of occluding or covering major arch vessels. Additionally, the aneurysmal degeneration of the left subclavian artery precluded a standard left carotid-subclavian bypass. These anatomic challenges required a strategic surgical plan to ensure effective aneurysm seal while preserving cerebral perfusion. 

Left carotid-subclavian bypass has been well-documented as an effective means of preserving perfusion before TEVAR [7]. However, in the setting of a left subclavian artery aneurysm or other vascular defects, alternative strategies must be considered. The use of a right-to-left carotid bypass via a retroesophageal tunnel, as performed in this case, is a less commonly reported technique but offers advantages in preserving cerebral perfusion, particularly when standard revascularization routes are not viable. A study performed by Ozsvath et al. examined 24 carotid-carotid crossover bypass procedures and concluded a three-year 92% patency rate with a four-year stroke-free survival rate of 94% [8]. Additionally, retroesophageal routing in a carotid-carotid bypass graft provides a shorter and more direct path between the carotid arteries, which can minimize kinking and redundancy, thereby reducing the risk of thrombosis. These findings support the viability of this approach for complex aortic reconstructions. 

Type 1A endoleaks occur due to inadequate sealing at the proximal attachment site of the endograft and present a significant risk for continued aneurysmal expansion and rupture. The incidence of type 1A endoleaks varies depending on the endovascular technique employed. A systematic review conducted by Scurto et al. revealed that type 1A endoleaks occurred at an 18.8% rate in chimney procedures, a 4.8% rate in fenestrated devices, 3% rate in branched devices, and a 2.2% rate in in situ fenestration [9]. This further exemplifies the challenges associated with achieving optimal proximal seal in the aortic arch.

Repair of type 1A endoleaks carries with it an overall mortality rate of 50% and an operative mortality rate of 41% [3]. This emphasizes the need for immediate repair and careful consideration of the operative approach. Traditional management for type 1A endoleaks involves endovascular interventions such as placement of an aortic extension cuff to extend the stent graft coverage. However, in this case, balloon-assisted fixation alone effectively resolved the endoleak without requiring additional stent placement. This highlights the fact that, in certain cases, satisfactory results can be achieved with less procedural complexity and reduced potential risk to the patient.

This case adds to the literature by emphasizing the importance of an individualized surgical plan in the management of complex TAA complicated by endoleaks. A thorough preoperative evaluation of the aortic arch and supra-aortic vessels, with CTA, is critical in determining the most appropriate revascularization strategy. The patient's advanced age and comorbidities also guided the decision toward a minimally invasive approach. While frailty assessment is often used in preoperative planning, it was not formally documented in this case. In cases where more proximal deployment of a TEVAR graft is required, a right-to-left carotid bypass via a retroesophageal tunnel and carotid subclavian transposition offers a viable alternative. Attempting less invasive methods, such as balloon-assisted fixation, can also be effective. Potentially reducing the need for additional endovascular devices and associated complications. 

## Conclusions

This case illustrates the importance of patient-specific surgical approaches when managing thoracic aortic aneurysms complicated by type 1A endoleak. A thorough preoperative assessment, including CTA for vascular anatomy and patient vulnerability assessments, should be performed to guide decision-making and improve surgical outcomes. This case contributes to the literature supporting hybrid and staged approaches in TEVAR, emphasizing the importance of tailored interventions in high-risk patients. Future studies should continue to evaluate the long-term outcomes of alternative revascularization techniques and endovascular management strategies for type 1A endoleaks.
